# Salutary Effects of Cepharanthine against Skeletal Muscle and Kidney Injuries following Limb Ischemia/Reperfusion

**DOI:** 10.1155/2015/504061

**Published:** 2015-10-26

**Authors:** Ming-Chang Kao, Chih-Yang Chung, Ya-Ying Chang, Chih-Kung Lin, Joen-Rong Sheu, Chun-Jen Huang

**Affiliations:** ^1^Graduate Institute of Medical Sciences, College of Medicine, Taipei Medical University, Taipei 11042, Taiwan; ^2^Department of Anesthesiology, Taipei Tzu Chi Hospital, New Taipei City 23142, Taiwan; ^3^School of Medicine, Tzu Chi University, Hualien 97004, Taiwan; ^4^Department of Pathology, Taipei Tzu Chi Hospital, New Taipei City 23142, Taiwan

## Abstract

Limb ischemia/reperfusion (I/R) causes oxidation and inflammation and subsequently induces muscle and kidney injuries. Cepharanthine, a natural plant alkaloid, possesses anti-inflammatory and antioxidative properties. We elucidated the salutary effects of cepharanthine against muscle and kidney injuries following limb I/R. Adult male rats were randomized to receive I/R or I/R plus cepharanthine. I/R was achieved by applying tourniquet high around each thigh for 3 hours followed by reperfusion for 24 hours. Cepharanthine (10 mg/kg, intraperitoneal) was injected immediately before reperfusion. After euthanization, degrees of tissue injury, inflammation, and oxidation were examined. Our data revealed that the I/R group had significant increases in injury biomarker concentrations of muscle (creatine kinase and lactate dehydrogenase) and kidney (creatinine, neutrophil gelatinase-associated lipocalin, and kidney injury molecule-1). Histological assays revealed moderate muscle and kidney injury characteristics in the I/R group. The I/R group also had significant increases in concentrations of inflammatory molecules (interleukin-6, macrophage inflammatory protein-2, and prostaglandin E_2_) and reactive nitrogen species (nitric oxide) as well as lipid peroxidation (malondialdehyde). Of note, these effects of limb I/R could be mitigated by cepharanthine. These data confirmed that cepharanthine attenuated muscle and kidney injuries induced by limb I/R. The mechanisms may involve its anti-inflammatory and antioxidative capacities.

## 1. Introduction

Limb ischemia/reperfusion (I/R) can be frequently encountered in clinical settings, including lower limb arterial surgery and critical limb ischemia and revascularization for thromboembolic events involving the lower extremities [1–5]. Limb I/R not only causes local skeletal muscle damage but may also lead to remote organ injury [6, 7]. With acute limb ischemia, increased anaerobic metabolism can lead to muscle acidosis, alteration of muscle cell permeability, and ultimately result in muscle damage and subsequent rhabdomyolysis [7, 8]. During reperfusion, restoration of blood supply to the ischemic muscle further activates inflammation and oxidative damage. Moreover, the acidic metabolites, proinflammatory cytokines, and large amounts of reactive oxygen and nitrogen species released from the damaged muscles may trigger systemic inflammatory response and multiple organ dysfunctions [6–8]. As a consequence, acute kidney injury is one of the most dreadful complications following limb I/R [8–12]. Therapies aimed at attenuating I/R-induced inflammatory response and oxidative stress may reduce the risk of acute kidney injury following limb I/R [9].

Cepharanthine is a biscoclaurine alkaloid isolated from a natural herb, Stephania cepharantha Hayata [13]. Cepharanthine has been widely used in clinical field for decades to treat a variety of acute and chronic diseases, such as radiation-induced leucopenia and alopecia [13]. Cepharanthine possesses anti-inflammatory, antioxidative, antiallergic, immunomodulatory, and many other beneficial biological activities [13, 14]. In the past decades, there is a growing interest in the protective effects of naturally occurring compounds on inflammatory disorders. Recent studies have demonstrated the beneficial effects of cepharanthine on several experimental models of inflammation [15–18]. Moreover, a recent study revealed that cepharanthine could attenuate acute kidney injury in a rat model of local renal I/R [19].

Limb I/R-induced skeletal muscle and kidney injuries are complex inflammatory disorder involving both local and remote organ dysfunction. To date, the question of whether cepharanthine can play a protective role in this situation remains unstudied. Therefore, we carried out this study to evaluate the possible salutary effects of cepharanthine against muscle and kidney injuries in a rat model of hind limb I/R.

## 2. Materials and Methods

### 2.1. Animal Preparation

A total of 48 male Sprague-Dawley rats (200 to 250 g; BioLASCO Taiwan Co., Ltd., Taipei, Taiwan) were used for the experiments. All animal studies were approved by the Institutional Animal Use and Care Committee, Taipei Tzu Chi Hospital (103-IACUC number 031). The care and handling of the animals were performed in accordance with the guidelines of the National Institutes of Health. All rats were anesthetized with intramuscular injection of a zoletil/xylazine mixture (30/10 mg/kg) and placed supine on a board. A rectal temperature probe was inserted and the body temperature was maintained at 37°C using the heating pad and heating lamps. Supplemental one-third of doses of zoletil/xylazine mixture were administered hourly until the end of each experiment.

### 2.2. Hind Limb I/R Model

The hind limb I/R protocol was adapted from previous reports [20, 21]. Ischemia was induced by applying rubber band tourniquet high around bilateral thighs for 3 hours and followed by removal of the rubber band tourniquet (i.e., reperfusion) for 24 hours. During reperfusion period, the rats were returned to their cages and allowed to access to a commercial balanced diet and water ad libitum.

### 2.3. Experimental Designs

The rats were randomly allocated to one of the four groups: the sham, the sham + CEP, the I/R, and the I/R + CEP groups (*n* = 12 per group). The sham group received sham operation plus a 30 *μ*L intraperitoneal injection (ip) of dimethylsulfoxide (DMSO, i.e., the vehicle; Sigma, St. Louis, MO, USA). The sham + CEP group received sham operation plus cepharanthine (10 mg/kg, ip; LKT Laboratories, Inc. St. Paul, MN, USA). The I/R group received I/R plus the vehicle. The I/R + CEP group received I/R plus cepharanthine (10 mg/kg, ip). Intraperitoneal injection of vehicle or cepharanthine was performed immediately before reperfusion or at comparable time point in the sham groups. The dose of cepharanthine was determined according to previous reports [15, 19].

### 2.4. Limb Perfusion Measurement

The rats were anesthetized at the end of reperfusion. Perfusion in the gracilis muscle of the right hind limb was measured with a laser-Doppler probe (ABLPHDI, Transonic Systems, Ithaca, NY, USA) connecting to a tissue perfusion monitor (BFL22, Transonic Systems). The tissue volume in the calculations was assumed to be 1 mm^3^ and the blood flow was analyzed using Doppler light shift from moving red blood cells by the Bonner algorithm [22]. The values were reported as tissue perfusion units (TPU), which were proportional to the absolute units (mL × min^−1^ × 100 g^−1^ of tissue) [23].

### 2.5. Plasma, Urine, and Tissue Sample Collection

After measuring the limb perfusion, a laparotomy was made. The blood sample was collected followed by plasma separation. The urine sample was collected from aspiration of the bladder. The plasma and urine samples were stored at −80°C for subsequent analysis. The rat was then sacrificed by aortal exsanguinations and the bilateral kidneys and gastrocnemius muscles were removed. The left kidney and gastrocnemius muscle were snap-frozen in liquid nitrogen and stored at −80°C until further analysis. The right kidney and gastrocnemius muscle were divided into two parts. One part was fixed in 10% formaldehyde for one day and then embedded in paraffin for histological analysis. The other half was used for wet/dry weight ratio measurement.

### 2.6. Plasma Laboratory Parameters

The levels of creatine kinase (CK) and lactate dehydrogenase (LDH) in plasma were measured using the DXC 800 general chemistry systems (Beckman Coulter, Brea, CA, USA) to assess muscle injury. The level of creatinine in plasma was also measured using the same instrument to measure kidney function.

### 2.7. Acute Kidney Injury Biomarkers

Plasma and urinary concentrations of novel acute kidney injury biomarkers, including neutrophil gelatinase-associated lipocalin (NGAL) and kidney injury molecule-1 (KIM-1), were measured using commercially available enzyme-linked immunosorbent assay (ELISA) test kits (R&D Systems, Minneapolis, MN, USA). Measurements of NGAL and KIM-1 were performed according to the manufacturer's protocols.

### 2.8. Histological Analysis and Wet/Dry Weight Ratio

The paraffin-embedded muscle and kidney samples were serial sectioned and stained with hematoxylin and eosin. Histological examinations were carried out using light microscope (200x) by a pathologist who was blinded to the experiment. Ten visual fields were randomly chosen to assess the degrees of tissue injury. For evaluation of muscle injury, the histological changes of mononuclear cell infiltration, interstitial edema, hemorrhage, and focal necrosis of the muscle tissues were assessed [24]. For evaluation of kidney injury, the histological changes of intracellular vacuolization, interstitial edema, cast formation, and tubular necrosis of the kidney tissues were assessed [25]. The tissue injury was further classified as normal, minimal, mild, moderate, or severe by the same pathologist. To further quantify the extent of muscle injury, the number of injured fibers was counted in 15 photographed fields in the muscle cross sections using a standardized method according to a previous report [26]. The muscle injury score was expressed as injured fibers/total fibers (%). Moreover, wet/dry weight ratio (i.e., water content) was assayed by a protocol we have previously reported [20]. In brief, the freshly harvested muscle and kidney tissues were weighed and then placed in the oven at 80°C. After 24 hours, they were weighed again and the values of wet/dry weight ratio were calculated.

### 2.9. Myeloperoxidase (MPO) Activity

MPO activity (i.e., activity of infiltrated leukocytes) was measured using a protocol we have previously reported [20]. The snap-frozen muscle and kidney tissues were homogenized, resuspended, sonicated, and centrifuged. The supernatant was collected and incubated in a water bath at 60°C for 2 hours. MPO activity was then measured using the myeloperoxidase fluorometric detection kit (Enzo Life Science, Plymouth Meeting, PA, USA).

### 2.10. Inflammatory Molecules

The muscle and kidney tissues were processed as we have previously described [20]. The concentrations of inflammatory molecules in muscle and kidney samples, including interleukin-6 (IL-6), macrophage inflammatory protein-2 (MIP-2), and prostaglandin E_2_ (PGE_2_), were measured using commercially available ELISA kits (Enzo Life Science, Farmingdale, NY, USA).

### 2.11. Nitric Oxide (NO) and Malondialdehyde (MDA)

The muscle and kidney tissues were processed as we have previously described [20]. Concentration of reactive nitrogen species was determined by measuring the concentrations of NO metabolites (i.e., nitrite and nitrate), using a colorimetric assay kit (Cayman Chemical, Ann Arbor, MI, USA). Lipid peroxidation status was determined by measuring the MDA concentrations using a thiobarbituric acid reactive substances assay kit (Cayman Chemical Company, Ann Arbor, MI, USA).

### 2.12. Biomarker of Thrombus-Induced Ischemia

The muscle tissues were processed as we have previously described [20]. The concentrations of vascular endothelial growth factor (VEGF) in muscle samples were measured using ELISA (rat VEGF DuoSet ELISA development system, R&D Systems). Results were normalized to protein concentrations.

### 2.13. Statistical Analysis

Data were shown as means ± standard deviations. One-way analysis of variance with Tukey post hoc test was used for multiple comparisons. The significance level was set at 0.05. All data were analyzed using SigmaPlot for windows (SPSS Scientific, Chicago, IL, USA).

## 3. Results

### 3.1. CK, LDH, and Creatinine Levels in Plasma

**Figure 1:**
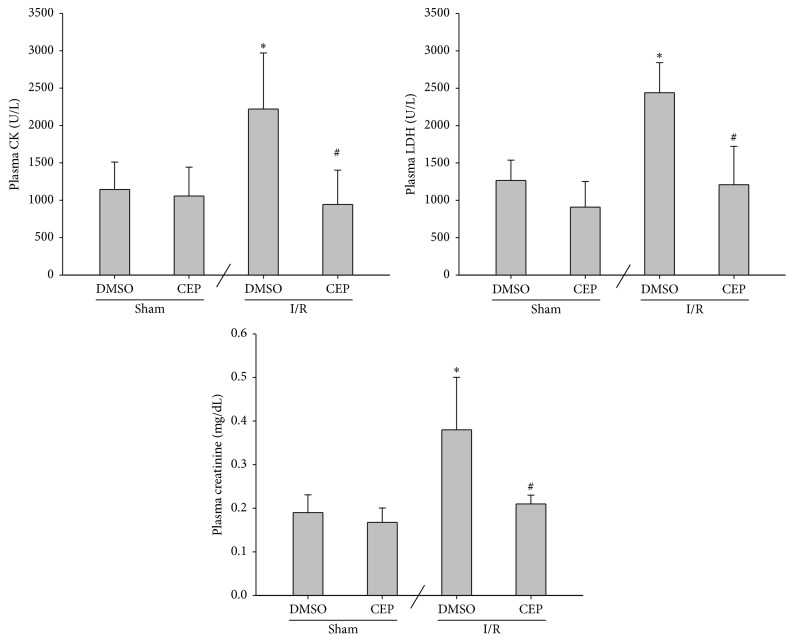
The plasma concentrations of creatine kinase (CK), lactate dehydrogenase (LDH), and creatinine. Sham: the sham group. Sham + CEP: the sham plus cepharanthine group. I/R: the limb ischemia-reperfusion group. I/R + CEP: the I/R plus cepharanthine group. DMSO: dimethylsulfoxide. Data were means ± standard deviations. ^*∗*^ *P* < 0.05* versus* the sham group. ^#^ *P* < 0.05, the I/R + CEP* versus* the I/R group.

The plasma CK concentrations of the sham and the sham + CEP groups were low (Figure 1). The plasma CK concentration of the I/R group was significantly higher than that of the sham group (*P* = 0.007; Figure 1). In contrast, the plasma CK concentration of the I/R + CEP group was significantly lower than that of the I/R group (*P* = 0.002; Figure 1).

The data of plasma LDH and creatinine essentially paralleled the data of CK (Figure 1).

### 3.2. NGAL and KIM-1 Levels in Plasma and Urine

**Figure 2:**
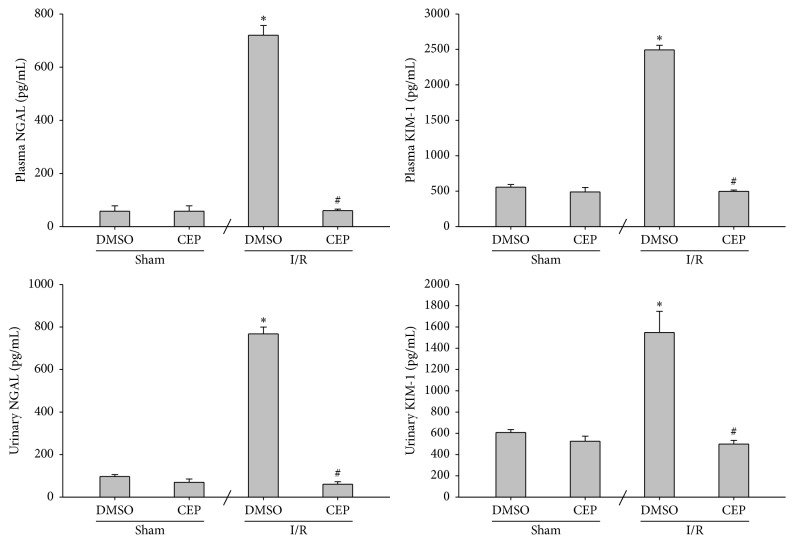
The plasma and urinary concentrations of neutrophil gelatinase-associated lipocalin (NGAL) and kidney injury molecule-1 (KIM-1). Sham: the sham group. Sham + CEP: the sham plus cepharanthine group. I/R: the limb ischemia-reperfusion group. I/R + CEP: the I/R plus cepharanthine group. DMSO: dimethylsulfoxide. Data were means ± standard deviations. ^*∗*^ *P* < 0.05* versus* the sham group. ^#^ *P* < 0.05, the I/R + CEP* versus* the I/R group.

The plasma and urinary NGAL concentrations of the sham and the sham + CEP groups were low (Figure 2). The plasma and urinary NGAL concentrations of the I/R group were significantly higher than those of the sham group (both *P* < 0.001; Figure 2). In contrast, the plasma and urinary NGAL concentrations of the I/R + CEP group were significantly lower than those of the I/R group (both *P* < 0.001; Figure 2).

Similarly, the data of plasma and urinary KIM-1 essentially paralleled the data of NGAL (Figure 2).

### 3.3. Tissue Perfusion in Hind Limb

**Figure 3:**
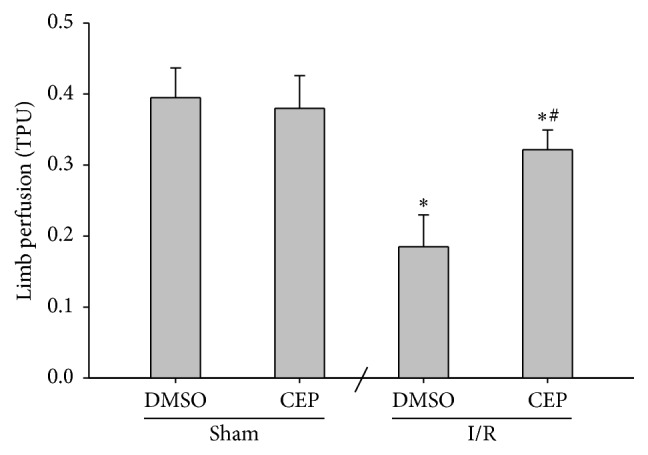
The limb perfusion in the gracilis muscle of the right hind limb. TPU: tissue perfusion units. Sham: the sham group. Sham + CEP: the sham plus cepharanthine group. I/R: the limb ischemia-reperfusion group. I/R + CEP: the I/R plus cepharanthine group. DMSO: dimethylsulfoxide. Data were means ± standard deviations. ^*∗*^ *P* < 0.05* versus* the sham group. ^#^ *P* < 0.05, the I/R + CEP* versus* the I/R group.

The tissue perfusions of the sham and the sham + CEP groups were high (Figure 3). The tissue perfusion of the I/R group was significantly lower than that of the sham group (*P* < 0.001; Figure 3). In contrast, the tissue perfusion of the I/R + CEP group was significantly higher than that of the I/R group (*P* < 0.001; Figure 3).

### 3.4. Histological Findings and Wet/Dry Weight Ratio in Muscle and Kidney

**Figure 4:**
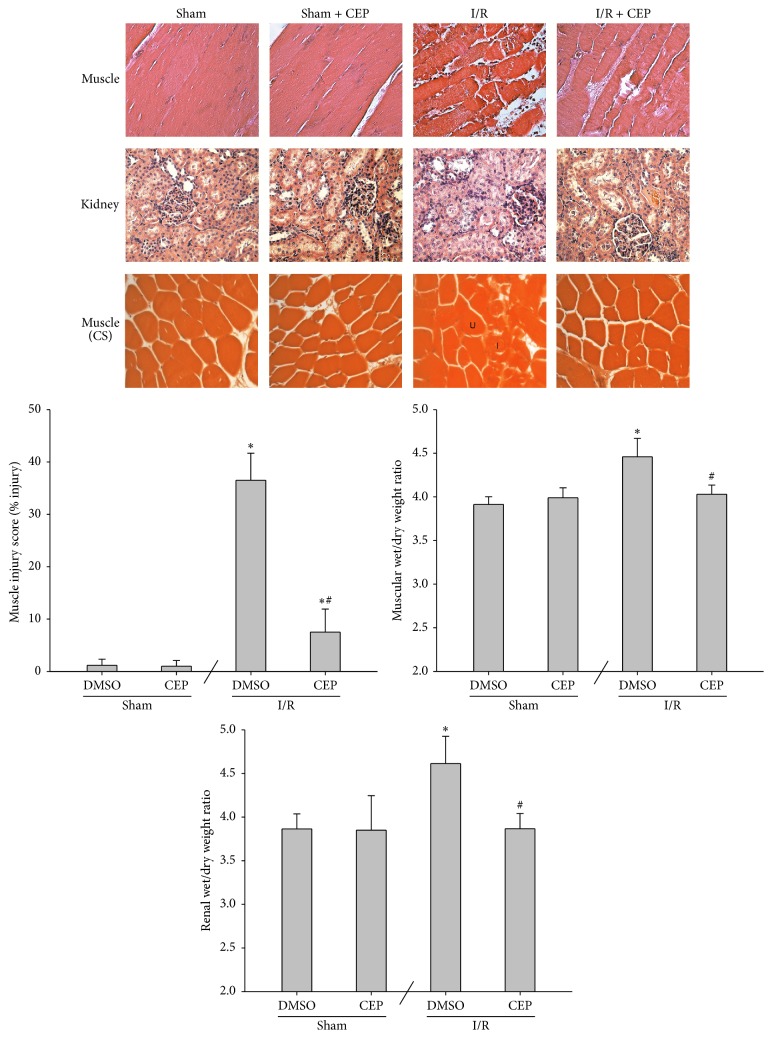
Representative microscopic findings of the muscle and kidney tissues stained with hematoxylin and eosin (200x). The muscle injury score (% injury of skeletal muscle fibers) and the wet/dry weigh ratio in muscle and kidney. Sham: the sham group. Sham + CEP: the sham plus cepharanthine group. I/R: the limb ischemia-reperfusion group. I/R + CEP: the I/R plus cepharanthine group. CS: cross section. U: uninjured muscle fiber. I: injured muscle fiber. DMSO: dimethylsulfoxide. Data were means ± standard deviations. ^*∗*^ *P* < 0.05* versus* the sham group. ^#^ *P* < 0.05, the I/R + CEP* versus* the I/R group.

The sham and the sham + CEP groups revealed normal morphology in the muscle and kidney tissues (Figure 4). In contrast, the I/R group revealed moderate injury histological changes in the muscle and kidney tissues (Figure 4). Moreover, the I/R + CEP group revealed minimal to mild injury histological changes in the muscle tissues and normal to minimal injury histological changes in the kidney tissues (Figure 4).

The muscle injury scores of the sham and the sham + CEP groups were low (Figure 4). The muscle injury scores of the I/R group were significantly higher than those of the sham group (*P* < 0.001; Figure 4). In contrast, the muscle injury score of the I/R + CEP group was significantly lower than that of the I/R group (*P* < 0.001; Figure 4).

The data of muscular and renal wet/dry weight ratio essentially paralleled the data of muscle injury score (Figure 4).

### 3.5. MPO Activity in Muscle and Kidney

**Figure 5:**
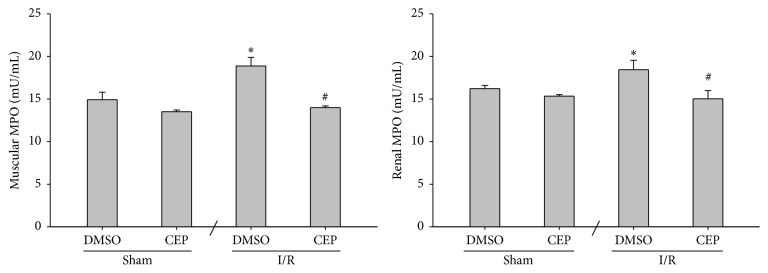
The myeloperoxidase (MPO) activity in muscle and kidney. Sham: the sham group. Sham + CEP: the sham plus cepharanthine group. I/R: the limb ischemia-reperfusion group. I/R + CEP: the I/R plus cepharanthine group. DMSO: dimethylsulfoxide. Data were means ± standard deviations. ^*∗*^ *P* < 0.05* versus* the sham group. ^#^ *P* < 0.05, the I/R + CEP* versus* the I/R group.

The muscular and renal MPO activities of the sham and the sham + CEP groups were low (Figure 5). The muscular and renal MPO activities of the I/R group were significantly higher than those of the sham group (*P* < 0.001 and =0.005, resp.; Figure 5). In contrast, the muscular and renal MPO activities of the I/R + CEP group were significantly lower than those of the I/R group (*P* < 0.001 and =0.003, resp.; Figure 5).

### 3.6. Inflammatory Molecules in Muscle and Kidney

**Figure 6:**
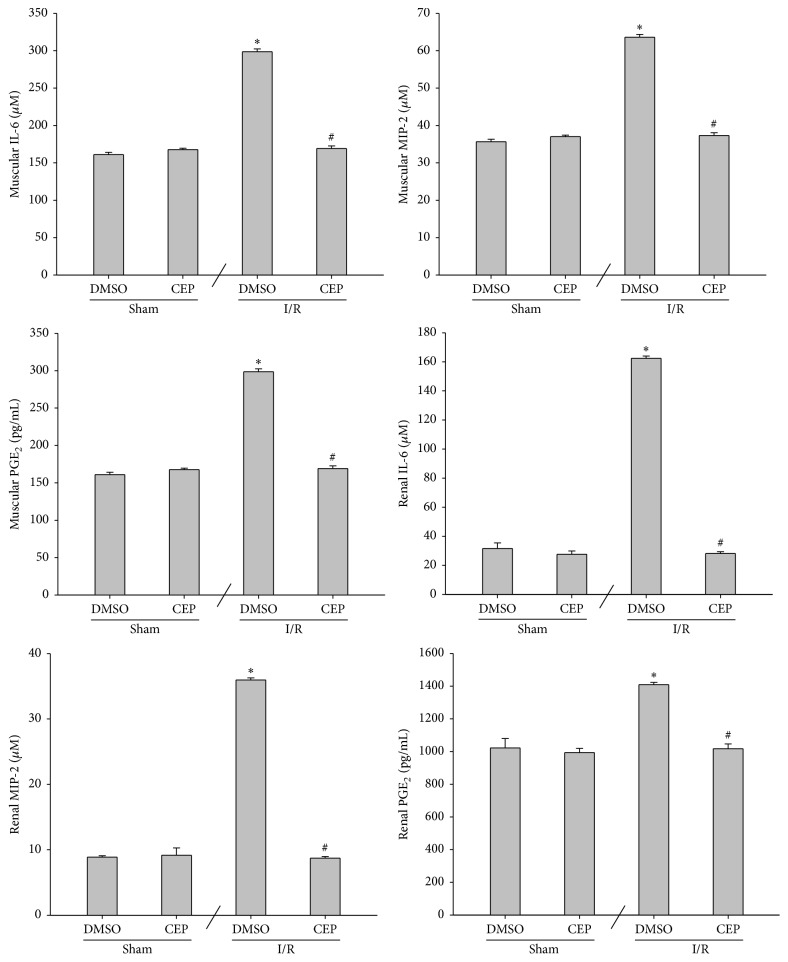
The concentrations of interleukin-6 (IL-6), macrophage inflammatory protein-2 (MIP-2), and prostaglandin E_2_ (PGE_2_) in muscle and kidney. Sham: the sham group. Sham + CEP: the sham plus cepharanthine group. I/R: the limb ischemia-reperfusion group. I/R + CEP: the I/R plus cepharanthine group. DMSO: dimethylsulfoxide. Data were means ± standard deviations. ^*∗*^ *P* < 0.05* versus* the sham group. ^#^ *P* < 0.05, the I/R + CEP* versus* the I/R group.

The muscular and renal IL-6 concentrations of the sham and the sham + CEP groups were low (Figure 6). The muscular and renal IL-6 concentrations of the I/R group were significantly higher than those of the sham group (both *P* < 0.001; Figure 6). In contrast, the muscular and renal IL-6 concentrations of the I/R + CEP group were significantly lower than those of the I/R group (both *P* < 0.001; Figure 6).

The data of muscular and renal MIP-2 and PGE_2_ essentially paralleled the data of IL-6 (Figure 6).

### 3.7. NO and MDA Concentrations in Muscle and Kidney

**Figure 7:**
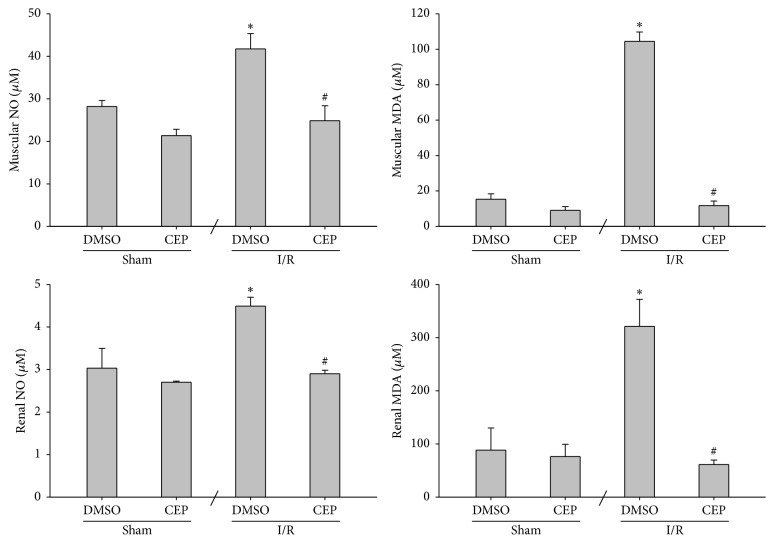
The concentrations of nitric oxide (NO) and malondialdehyde (MDA) in muscle and kidney. Sham: the sham group. Sham + CEP: the sham plus cepharanthine group. I/R: the limb ischemia-reperfusion group. I/R + CEP: the I/R plus cepharanthine group. DMSO: dimethylsulfoxide. Data were means ± standard deviations. ^*∗*^ *P* < 0.05* versus* the sham group. ^#^ *P* < 0.05, the I/R + CEP* versus* the I/R group.

The muscular and renal NO concentrations of the sham and the sham + CEP groups were also low (Figure 7). The muscular and renal NO concentrations of the I/R group were significantly higher than those of the sham group (both *P* < 0.001; Figure 7). In contrast, the muscular and renal NO concentrations of the I/R + CEP groups were significantly lower than those of the I/R group (both *P* < 0.001; Figure 7).

The data of muscular and renal MDA also paralleled the data of NO (Figure 7).

### 3.8. VEGF Concentrations in Muscle

**Figure 8:**
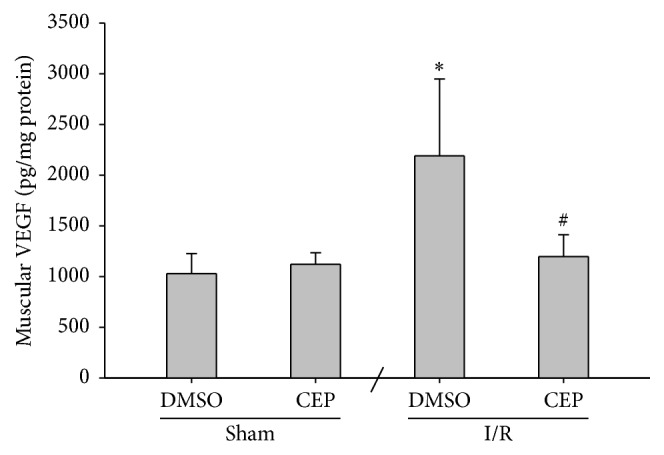
The concentrations of vascular endothelial growth factor (VEGF) in muscle. Sham: the sham group. Sham + CEP: the sham plus cepharanthine group. I/R: the limb ischemia-reperfusion group. I/R + CEP: the I/R plus cepharanthine group. DMSO: dimethylsulfoxide. Data were means ± standard deviations. ^*∗*^ *P* < 0.05* versus* the sham group. ^#^ *P* < 0.05, the I/R + CEP* versus* the I/R group.

The muscular VEGF concentrations of the sham and the sham + CEP groups were low (Figure 8). The muscular VEGF concentrations of the I/R group were significantly higher than those of the sham group (*P* = 0.045; Figure 8). In contrast, the muscular VEGF concentrations of the I/R + CEP group were significantly lower than those of the I/R group (*P* = 0.011; Figure 8).

## 4. Discussion

In this study, we used a rat model of hind lower limb I/R to assess the salutary effects of cepharanthine against muscle and kidney injuries induced by limb I/R. Our results are consistent with previous findings that limb I/R causes local skeletal muscle damage as well as remote acute kidney injury [8–12]. Specifically, our results confirmed that cepharanthine could attenuate the muscle and kidney injuries induced by limb I/R. Moreover, our findings indicated that the protective effects of cepharanthine may involve inhibition of inflammatory response and oxidative stress in both muscle and kidney.

Skeletal muscle is highly susceptible to ischemic insult and irreversible muscle damage may develop if focal limb ischemia exceeds 3 hours [7]. Reperfusion of the limbs may cause further muscle damage and release of myoglobin, CK, and other intracellular muscle contents, which may in turn result in acute kidney injury [11, 12]. Certain biochemical markers provide rapid and accurate assessment of the severity of organ injury and are readily available in the clinical laboratory setting. CK and LDH levels are two major blood chemical markers of muscle damage [9, 27]. Specifically, CK is a more sensitive indicator of skeletal muscle injury and predictor of renal failure than myoglobin [4]. For assessment of kidney injury, creatinine is a marker of renal function rather than injury and usually exhibits a delayed rise after injury occurs. In contrast, NGAL is a biomarker for ischemic injury [28] and KIM-1 is a biomarker for postischemic injury [29]. NGAL and KIM-1 are considered as the predictive markers of early acute kidney injury [30].

Data from this study confirmed that rats receiving limb I/R had significant increases in plasma levels of CK and LDH, indicating that I/R caused significant skeletal muscle injury. Moreover, our data demonstrated that rats receiving limb I/R had significant increases in plasma level of creatinine as well as plasma and urinary levels of NGAL and KIM-1, indicating that I/R caused significant acute kidney injury. In addition to biomarkers, our histological data confirmed that limb I/R could induce significant injury to the muscle tissues as the histological findings of the muscle tissues in the I/R group showed noticeable mononuclear cell infiltration, interstitial edema, hemorrhage, and muscle fiber necrosis changes [9, 31]. Our histological data also confirmed that limb I/R induced acute kidney injury, as the histological findings of the kidney tissues in the I/R group showed vacuolization, interstitial edema, cast formation, and tubular necrosis changes [4, 9]. The quantitative data of the muscle injury score as well as the wet/dry weight ratio in muscle and kidney further supported the histological findings in this study.

Of note, our data revealed that administration of cepharanthine immediately before reperfusion could inhibit the rises of all the biomarkers as well as the histological changes of the muscle and kidney tissues induced by limb I/R. These data provide clear evidence to support the concept that cepharanthine could mitigate the muscle and kidney injuries induced by limb I/R. It is established that inflammation and oxidation play crucial roles in mediating the muscle and kidney injuries induced by limb I/R [6–9]. Data from this study confirmed that rats receiving limb I/R had significant increases in concentrations of inflammatory molecules and reactive nitrogen species as well as lipid peroxidation in both the muscle and kidney tissues. Cepharanthine possesses potent anti-inflammatory and antioxidative effects [13, 14] and our data revealed that cepharanthine could inhibit the inflammation and oxidation induced by limb I/R. Judging from these data, we thus speculate that cepharanthine may very likely act through its anti-inflammatory and antioxidative effects to exert its salutary effects against the muscle and kidney injuries induced by limb I/R.

Prompt surgical or endovascular revascularization and pharmacological anticoagulation remain the keystone to restore blood flow into the ischemic limbs in clinical settings [32, 33]. Nevertheless, adjunctive pharmacotherapy with anti-inflammatory and antioxidative agents is crucial to decrease reperfusion injury in ischemic limbs [34]. Comparing to the I/R group, our data revealed that limb perfusion of the I/R + CEP group was significantly improved at 24 hours after reperfusion. Our data also revealed that the increase in muscular VEGF (an ischemia-sensitive marker that elevates in thrombus-induced limb ischemia) [35] was also inhibited by cepharanthine. Together, these data indicate that, in addition to its antioxidative and anti-inflammatory effects, cepharanthine may also act through improving limb circulation and mitigate vascular thrombosis to exert its therapeutic effects against limb I/R.

However, the mechanism underlying the effects of circulation improvement and vascular thrombosis mitigation induced by cepharanthine remains to be elucidated. Muscular PGE_2_ is a potent modulator of inflammation [36]. PGE_2_ per se can be proinflammatory that promotes local vasodilatation and activation of neutrophils, macrophages, and mast cells at early stage of inflammation [37]. In contrast, PGE_2_ also plays an important role in muscle healing. Upregulation of PGE_2_ signaling has been reported to regulate myogenesis by promoting myoblast proliferation via the PGE_2_ receptor 4 receptor [33]. Our data revealed that muscular PGE_2_ increased after limb I/R. However, limb I/R decreased limb circulation and increased inflammation in rats. These data indicated that the net effects of the muscular PGE_2_ increase induced by limb I/R were mainly proinflammatory and the vasodilation effects induced by muscular PGE_2_ were negligible. Moreover, cepharanthine could inhibit the increase of muscular PGE_2_ induced by limb ischemia. These data provide clear evidence to indicate that cepharanthine can inhibit the PGE_2_-related vasodilatation. However, considering the fact that cepharanthine could improve limb circulation in limb I/R rats, we thus speculate that the mechanisms underlying the effects of cepharanthine on increasing limb circulation after limb I/R should also be related to its anti-inflammatory and antioxidation effects.

Certain limitations in this study need to be addressed. Firstly, the protective effects of cepharanthine against muscle and kidney injuries induced by limb I/R involved inhibition of inflammation and oxidation. However, the underlying signaling pathways remain unstudied. Secondly, we used a single dose of cepharanthine in this study and examined the protective effects at 24 hours after reperfusion. Thus, the long-term outcome and the clinical application of this drug require further studies. Thirdly, the current study emphasized the protective effects of cepharanthine on injury biomarkers and histological analysis. Future investigation into its role on energy metabolism is needed before further conclusion can be drawn in this regard.

In summary, cepharanthine significantly attenuated local skeletal muscle damage and prevented remote kidney injury induced by bilateral hind limb I/R in rats. The mechanisms may involve its effects on inhibiting inflammation and oxidation.

## References

[1] Groeneveld A. B. J., Raijmakers P. G. H., Rauwerda J. A., Hack C. E. (1997). The inflammatory response to vascular surgery-associated ischaemia and reperfusion in man: effect on postoperative pulmonary function. *European Journal of Vascular and Endovascular Surgery*.

[2] Paterson I. S., Klausner J. M., Pugath R. (1989). Noncardiogenic pulmonary edema after abdominal aortic aneurysm surgery. *Annals of Surgery*.

[3] Volanska M., Zavacky P., Bober J. (2006). Ischaemic-reperfusion damage of tissue and critical limb ischaemia. *Bratislavské Lekárske Listy*.

[4] Eliason J. L., Wakefield T. W. (2009). Metabolic consequences of acute limb ischemia and their clinical implications. *Seminars in Vascular Surgery*.

[5] Szijártó A., Turóczi Z., Szabó J. (2013). Rapidly progressing fatal reperfusion syndrome caused by acute critical ischemia of the lower limb. *Cardiovascular Pathology*.

[6] Yassin M. M. I., Harkin D. W., Barros D'Sa A. A. B., Halliday M. I., Rowlands B. J. (2002). Lower limb ischemia-reperfusion injury triggers a systemic inflammatory response and multiple organ dysfunction. *World Journal of Surgery*.

[7] Blaisdell F. W. (2002). The pathophysiology of skeletal muscle ischemia and the reperfusion syndrome: a review. *Cardiovascular Surgery*.

[8] Goksin I., Adali F., Enli Y. (2011). The effect of phlebotomy and mannitol on acute renal injury induced by ischemia/reperfusion of lower limbs in rats. *Annals of Vascular Surgery*.

[9] Garbaisz D., Turoczi Z., Aranyi P. (2014). Attenuation of skeletal muscle and renal injury to the lower limb following ischemia-reperfusion using mPTP inhibitor NIM-811. *PLoS ONE*.

[10] Neto A. A. M., Júnior S. S. D. S., Capelozzi V. L. (2012). Effects of cilostazol in kidney and skeletal striated muscle of Wistar rats submitted to acute ischemia and reperfusion of hind limbs. *Acta Cirurgica Brasileira*.

[11] Takhtfooladi M. A., Takhtfooladi H. A., Moayer F., Karimi P., Asl H. A. (2015). Effect of *Otostegia persica* extraction on renal injury induced by hindlimb ischemia-reperfusion: a rat model. *International Journal of Surgery*.

[12] Adiseshiah M., Round J. M., Jones D. A. (1992). Reperfusion injury in skeletal muscle: a prospective study in patients with acute limb ischaemia and claudicants treated by revascularization. *British Journal of Surgery*.

[13] Rogosnitzky M., Danks R. (2011). Therapeutic potential of the biscoclaurine alkaloid, cepharanthine, for a range of clinical conditions. *Pharmacological Reports*.

[14] Furusawa S., Wu J. (2007). The effects of biscoclaurine alkaloid cepharanthine on mammalian cells: implications for cancer, shock, and inflammatory diseases. *Life Sciences*.

[15] Kudo K., Hagiwara S., Hasegawa A., Kusaka J., Koga H., Noguchi T. (2011). Cepharanthine exerts anti-inflammatory effects via NF-*κ*B inhibition in a LPS-induced rat model of systemic inflammation. *Journal of Surgical Research*.

[16] Huang H., Hu G., Wang C., Xu H., Chen X., Qian A. (2014). Cepharanthine, an alkaloid from Stephania cepharantha Hayata, inhibits the inflammatory response in the RAW264.7 cell and mouse models. *Inflammation*.

[17] Murakami K., Cox R. A., Hawkins H. K. (2003). Cepharanthin, an alkaloid from *Stephania cepharantha*, inhibits increased pulmonary vascular permeability in an ovine model of sepsis. *Shock*.

[18] Ershun Z., Yunhe F., Zhengkai W., Yongguo C., Naisheng Z., Zhengtao Y. (2014). Cepharanthine attenuates lipopolysaccharide-induced mice mastitis by suppressing the NF-*κ*B signaling pathway. *Inflammation*.

[19] Kusaka J., Hagiwara S., Hasegawa A., Kudo K., Koga H., Noguchi T. (2011). Cepharanthine improves renal ischemia-reperfusion injury in rats. *Journal of Surgical Research*.

[20] Kao M.-C., Jan W.-C., Tsai P.-S., Wang T.-Y., Huang C.-J. (2011). Magnesium sulfate mitigates lung injury induced by bilateral lower limb ischemia-reperfusion in rats. *Journal of Surgical Research*.

[21] Duehrkop C., Rieben R. (2014). Refinement of tourniquet-induced peripheral ischemia/reperfusion injury in rats: comparison of 2 h vs 24 h reperfusion. *Laboratory Animals*.

[22] Suchkova V. N., Baggs R. B., Francis C. W. (2000). Effect of 40-kHz ultrasound on acute thrombotic ischemia in a rabbit femoral artery thrombosis model: enhancement of thrombolysis and improvement in capillary muscle perfusion. *Circulation*.

[23] Guzman J. A., Rosado A. E., Kruse J. A. (2003). Dopamine-1 receptor stimulation impairs intestinal oxygen utilization during critical hypoperfusion. *American Journal of Physiology—Heart and Circulatory Physiology*.

[24] Duehrkop C., Denoyelle J., Shaw S., Rieben R. (2014). Use of dextran sulfate in tourniquet-induced skeletal muscle reperfusion injury. *Journal of Surgical Research*.

[25] Tan S., Wang G., Guo Y., Gui D., Wang N. (2013). Preventive effects of a natural anti-inflammatory agent, astragaloside IV, on ischemic acute kidney injury in rats. *Evidence-Based Complementary and Alternative Medicine*.

[26] McCormack M. C., Kwon E., Eberlin K. R. (2008). Development of reproducible histologic injury severity scores: skeletal muscle reperfusion injury. *Surgery*.

[27] Takhtfooladi H. A., Takhtfooladi M. A., Karimi P., Asl H. A., Mobarakeh S. Z. M. N. (2014). Influence of tramadol on ischemia-reperfusion injury of rats' skeletal muscle. *International Journal of Surgery*.

[28] Mishra J., Mori K., Ma Q. (2004). Amelioration of ischemic acute renal injury by neutrophil gelatinase-associated lipocalin. *Journal of the American Society of Nephrology*.

[29] Han W. K., Bailly V., Abichandani R., Thadhani R., Bonventre J. V. (2002). Kidney Injury Molecule-1 (KIM-1): a novel biomarker for human renal proximal tubule injury. *Kidney International*.

[30] Honore P. M., Jacobs R., Joannes-Boyau O. (2012). Biomarkers for early diagnosis of AKI in the ICU: ready for prime time use at the bedside?. *Annals of Intensive Care*.

[31] Huang T., Wang W., Tu C., Yang Z., Bramwell D., Sun X. (2015). Hydrogen-rich saline attenuates ischemia-reperfusion injury in skeletal muscle. *Journal of Surgical Research*.

[32] Mangiafico R. A., Mangiafico M. (2011). Medical treatment of critical limb ischemia: current state and future directions. *Current Vascular Pharmacology*.

[33] Mo C., Zhao R., Vallejo J. (2015). Prostaglandin E2 promotes proliferation of skeletal muscle myoblasts via EP4 receptor activation. *Cell Cycle*.

[34] Gulati A., Botnaru I., Garcia L. A. (2015). Critical limb ischemia and its treatments: a review. *The Journal of Cardiovascular Surgery*.

[35] Lee J.-H. (2012). A new rat pain model of thrombus-induced ischemia. *Methods in Molecular Biology*.

[36] Prisk V., Huard J. (2003). Muscle injuries and repair: the role of prostaglandins and inflammation. *Histology and Histopathology*.

[37] Kalinski P. (2012). Regulation of immune responses by prostaglandin E_2_. *Journal of Immunology*.

